# Enhanced effect of combining bone marrow mesenchymal stem cells (BMMSCs) and pulsed electromagnetic fields (PEMF) to promote recovery after spinal cord injury in mice

**DOI:** 10.1002/mco2.160

**Published:** 2022-08-03

**Authors:** Liyi Huang, Xin Sun, Lu Wang, Gaiqing Pei, Yang Wang, Qing Zhang, Zejun Liang, Dong Wang, Chenying Fu, Chengqi He, Quan Wei

**Affiliations:** ^1^ Rehabilitation Medicine Center and Institute of Rehabilitation Medicine, West China Hospital Sichuan University Chengdu PR China; ^2^ Key Laboratory of Rehabilitation Medicine in Sichuan Province Sichuan University Chengdu PR China; ^3^ National Clinical Research Center for Geriatrics, West China Hospital Sichuan University Chengdu Sichuan PR China; ^4^ Aging and Geriatric Mechanism Laboratory, West China Hospital Sichuan University Chengdu Sichuan PR China

**Keywords:** bone marrow mesenchymal stem cells, motor functional recovery, nutritional factors, pulsed electromagnetic fields, spinal cord injury

## Abstract

Spinal cord injury (SCI) is a destructive traumatic disease of the central nervous system without satisfying therapy efficiency. Bone marrow mesenchymal stem cells (BMMSCs) therapy promotes the neurotrophic factors’ secretion and axonal regeneration, thereby promoting recovery of SCI. Pulsed electromagnetic fields (PEMF) therapy has been proven to promote neural growth and regeneration. Both BMMSCs and PEMF have shown curative effects for SCI; PEMF can further promote stem cell differentiation. Thus, we explored the combined effects of BMMSCs and PEMF and the potential interaction between these two therapies in SCI. Compared with the SCI control, BMMSCs, and PEMF groups, the combinational therapy displayed the best therapeutic effect. Combinational therapy increased the expression levels of nutritional factors including brain‐derived neurotrophic factor (BDNF), nerve growth factors (NGF) and vascular endothelial growth factor (VEGF), enhanced neuron preservation (NeuN and NF‐200), and increased axonal growth (MBP and myelin sheath). Additionally, PEMF promoted the expression levels of BDNF and VEGF in BMMSCs via Wnt/β‐catenin signaling pathway. In summary, the combined therapy of BMMSCs and PEMF displayed a more satisfactory effect than BMMSCs and PEMF therapy alone, indicating a promising application of combined therapy for the therapy of SCI.

## Graphical abstract

The combination of bone marrow mesenchymal stem cells (BMMSCs) and pulsed electromagnetic fields (PEMF) improved the functional recovery in SCI mice by decreasing glial scar area, promoting the expression levels of nutritional factors, enhancing neuron preservation and axonal regeneration, which may be achieved by modulating Wnt/β‐catenin signaling pathway.

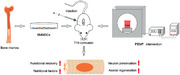

## 1 INTRODUCTION

Spinal cord injury (SCI) is a disastrous disability‐causing disease.^1^ It is also one of the most serious public health issues that causes heavy burdens for both the individual and overall society because of its high disability rate and high medical cost.^2^ A total of 3,000,000 SCI survivors live with severe disability and poor quality of life worldwide.^1^ They usually had paraplegia or tetraplegia, loss of sensory/motor function, urinary and bowel disorders, and other systemic complications. Injury of the spinal cord immediately results in loss of neurons, axonal membrane disruption, and rupture of nerve fibers. Besides, a series of secondary events, including an inflammatory response, inhibitory microenvironments, and glial scarring, further exacerbates the physiological function of patients with SCI.^3^ Unfortunately, current clinical treatments have limited efficacy, and the restoration of function after SCI remains an elusive goal.^4^ Since SCI is a multifactorial syndrome, it is paramount to hunt for therapies that play multiple roles.

In recent years, cell therapies have raised the hopes for SCI recovery due to their roles in nutritional support, neuroprotection, and nerve regeneration.^1^ Bone marrow mesenchymal stem cells (BMMSCs) derived from bone marrow primarily maintain bone regeneration and hematopoiesis^5^ are among the most promising cell candidates for therapeutic use because they are ethical to use, relatively easy to obtain, have low immunogenic effects and high satisfactory therapeutic effects.^6^ Up to now, more than 20 clinical trials have investigated the effects of BMMSCs on SCI recovery. Some completed clinical trials showed that BMMSCs improved SCI motor and sensory score, electromyography (EMG), somatosensory evoked potential (SEP) and other evaluation scales. Treating with BMMSCs has a variety of benefits, including promoting axonal regeneration, alleviating glial scar formation, inhibiting overactive inflammation, etc.^7^, ^8^ However, there are still lots of limitations for the wide application of BMMSCs because of the low survival ability, low proliferation potential, and unsatisfying homing ability to the injury site.^9^, ^10^, ^11^, ^12^, ^13^ More and more research focus on combinational therapy of BMMSCs and other therapy to provide better therapeutic efficiency for SCI.

It is reported that pulsed electromagnetic fields (PEMF) expedite growth and regeneration of neurons, and thus stimulate peripheral nerves functional recovery.^14^, ^15^, ^16^ In the central nervous system, PEMF also promote neurite outgrowth from spinal neurons and dorsal root ganglions.^15^ Some animal experiments suggested that PEMF intervention facilitated locomotion and sensory recovery in SCI models by enhancing neurite outgrowth and blood flow, downregulating inflammation and hazardous substances,^17^, ^18^, ^19^ as well as reducing muscle degeneration.^20^, ^21^, ^22^, ^23^, ^24^ Besides, electromagnetic fields (EMF) facilitate the recovery of SCI animals by promoting axonal regeneration and axon function.^25^ It also possesses positive effects on osteoblast proliferation and regeneration after SCI.^26^, ^27^ However, the therapeutic use of PEMF on SCI is still in its infancy.

Previous studies of BMMSCs and PEMF have shown that both therapies have therapeutic effects on SCI; however, the single therapy only achieves limited therapeutic efficacy due to its own limitation.^28^ Interestingly, PEMF has been proven to accelerate mesenchymal stem cell differentiation by regulating the Ca^2+^ ion channel.^29^ It is unclear whether the combination of these two therapies has a synergistic effect and interaction effect on the treatment of SCI. Therefore, we compared the treatment effect of BMMSCs, PEMF, and the combination of BMMSCs and PEMF on SCI mice model and explored the effects of PEMF on BMMSCs in vitro, to investigate (1) whether the combination of these two therapies is better than one single therapy and the potential molecular mechanism and (2) whether PEMF have an impact on BMMSCs and the underlying mechanism.

## 2 RESULTS

### 2.1 BMMSCs and PEMF promote locomotor functional recovery and reduce injury volume

After the initial isolation process, BMMSCs appeared as suspended round cells. At days 2–3, BMMSCs were observed as single cells or cell aggregates with polygonal, fusiform or star‐like structures. After passage 3, BMMSCs mainly presented with a spindle‐shaped morphology which conformed to the characteristics of BMMSCs (Figure S1A). The results of flow cytometry showed that the relative quantity of the surface markers CD29, CD34, CD44, and CD45 were 95.25%, 0.09%, 99.98%, and 1.00%, respectively (Figure S1B), which were all in line with the BMMSCs features.^30^, ^31^ The results of Alizarin red staining suggested that the cells had the ability to undergo osteogenic differentiation (Figure S1C). These results suggested that we successfully isolated and cultured BMMSCs.

**FIGURE 1:**
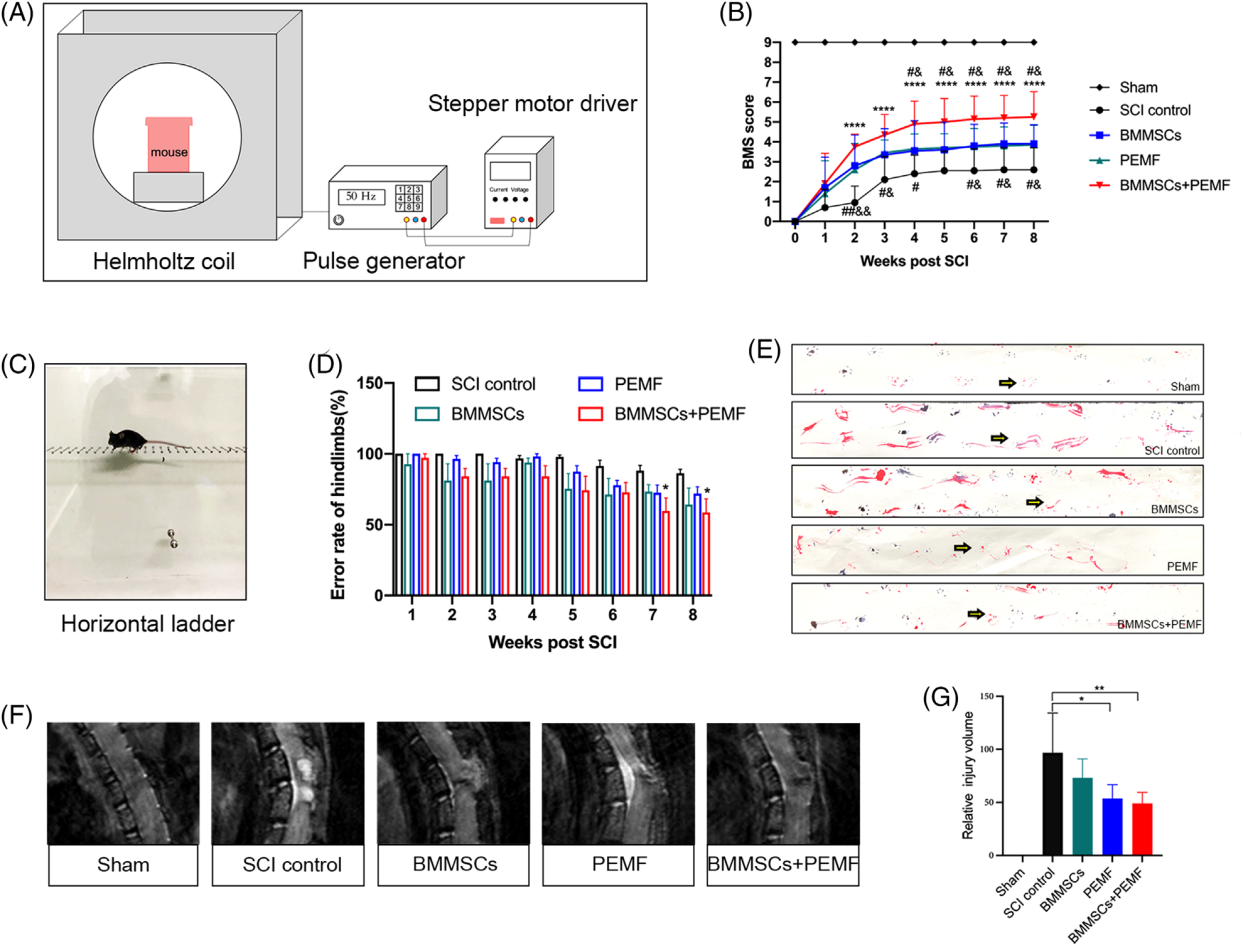
BMMSCs and PEMF promote functional recovery and reduce the injury volume in SCI mice. (A) Schematics of PEMF intervention in vivo. (B) Hindlimb locomotion after contusion injury in mice of the sham, SCI control, BMMSCs, PEMF, and BMMSCs+PEMF groups evaluated weekly from 1 to 56 days using the Basso Mouse Score (BMS) (*n* = 10). (C, D) Photograph of regular horizontal ladder (C) and quantification of error rate of hindlimbs. There was significant difference between SCI control and BMMSCs+PEMF at week 7 and week 8 (*n* = 5). (E) Representative footprint analysis images of the sham, SCI control, BMMSCs, PEMF, and BMMSCs+PEMF groups at week 8. (F, G) Sagittal images of the T10 injury epicenter were obtained by MRI at week 8. PEMF and BMMSCs+PEMF statistically decreased the injury volume compared with SCI control group (*n* = 7). (Error bars show mean ± SD; *p < 0.05, **p < 0.01, ***p < 0.001, ****p < 0.0001 compared with SCI control, ^#^ *p <* 0.05, compared with BMMSCs group, ^&^ *p <* 0.05, compared with PEMF group.)

**FIGURE 2:**
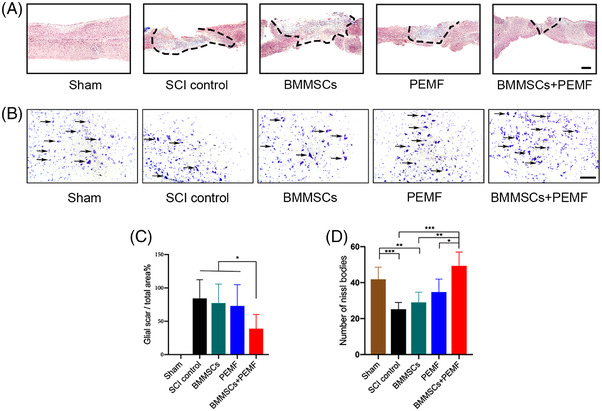
BMMSCs and PEMF reduce the injury volume and increase Nissl bodies in vivo. (A) Representative images of fibrous scar formation in the different groups. Area of fibrous scar was indicated by black dotted line (scale bar 200 μm). (B) Representative images of Nissl bodies in the different groups. Representative Nissl bodies were indicated with arrows (Scale bar 100 μm). (C) Percentage of fibrous scar area in the BMMSCs+PEMF group was significantly decreased compared to SCI control group. (D) Combination of BMMSCs and PEMF statistically increased the number of Nissl bodies compared to SCI control group, PEMF group and BMMSCs group. (Error bars show mean ± SD; *p < 0.05, **p < 0.01, ***p < 0.001.)

During treatments after SCI, the BMS score was utilized to evaluate the behavioral function recovery in mice. BMS scores were all zero after SCI model, while mice in the sham group maintained normal locomotor function. During the experimental period, mice in the PEMF, BMMSCs, and BMMSCs+PEMF groups obtained higher scores than SCI control group. Especially the mice in the BMMSCs+PEMF group exhibited the highest BMS score among groups, indicating the best locomotor recovery. BMS score of BMMSCs+PEMF group statistically raised from the 2nd week to the 8th week compared with that of SCI control group (*p *< 0.0001; Figure 1B). We also observed significant difference between SCI control group and single‐therapy group (*p *< 0.05). Importantly, BMS score in the BMMSCs+PEMF group significantly increased from the 4th week to the 8th week as compared to that in the PEMF group (*p *< 0.05). The same difference was also observed between the BMMSCs+PEMF group and BMMSCs group (*p *< 0.05). For the horizontal ladder analysis, there was significant difference between SCI control and BMMSCs+PEMF group at the 7th week and the 8th week (Figure 2C and D). Additionally, footprint analysis was used to further observe locomotor function. The results showed that the footprints of the mice in the sham group were coordinated, those in the SCI control group were trailed considerably, those in the PEMF and BMMSCs groups were relatively harmonious, and those in the BMMSCs+PEMF group represented the best gait with base of support (Figure 1E). As shown in Figure 1F and G, MRI images were used to evaluate the extent of the injury by volume at the end of the treatment duration. PEMF intervention decreased the injury volume compared to SCI control group (*p *< 0.05). BMMSCs+PEMF demonstrated a further decreasing trend compared with PEMF and displayed the smallest injury volume among all groups, showing significant difference compared with SCI control group (*p *< 0.01). These results indicating that these mice in the BMMSCs+PEMF group had the best locomotor recovery potential.

### 2.2 BMMSCs and PEMF decreased the fibrous scar formation and improve the expression of nutritional cytokines

At acute/subacute stages of SCI, glia scar inhibits further expansion of lesion site and preserves normal tissue. More importantly, the glia scar forms chemical and physical barriers to axon growth, exhibiting detrimental influence for recovery of SCI. We observed that fibrous scar area in the SCI control, BMMSCs and PEMF group were relatively larger than that in the BMMSCs+PEMF group (Figure 2A). BMMSCs or PEMF alone did not have any beneficial effects on the fibrous scar formation, whereas a significant lower fibrous scar area was observed in BMMSCs+PEMF group compared to any other groups (*p *< 0.05; Figure 2C). From the results of transverse sections, we found that Nissl bodies of spinal cord anterior horn decreased after SCI and increased after different interventions. Single therapy did not exhibit significant effect on number of Nissl bodies compared with SCI control group, while there was a remarkable difference between combinational therapy and SCI control group (*p *< 0.001, Figure 2B and D).

**FIGURE 3:**
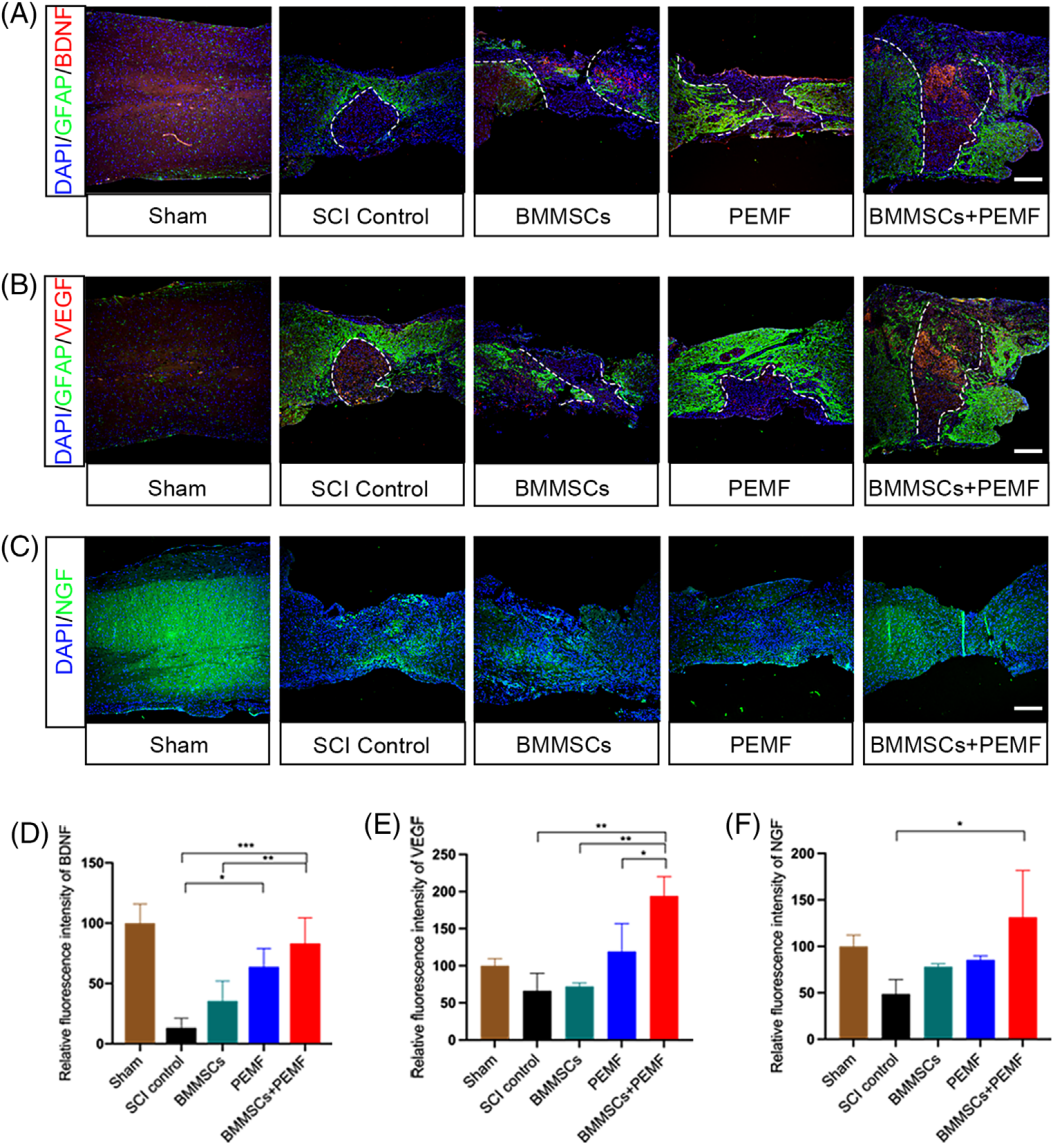
BMMSCs and PEMF increase the expression levels of BDNF, NGF and VEGF in vivo. (A) Representative immunofluorescence images of BDNF (red), astroglial maker GFAP (green), and DAPI (blue) in spinal cord lesions at week 8. (B) Representative immunofluorescence images of VEGF (red), astroglial maker GFAP (green) and DAPI (blue) in spinal cord lesions at week 8. (C) Representative immunofluorescence images of NGF (green) and DAPI (blue) in spinal cord lesions at week 8. (D) Quantification of the relative intensity of BDNF showed BMMSCs+PEMF group shows highest expression level of BDNF and had significant difference compared with SCI control group and BMMSCs group. PEMF group also increases BDNF expression compared with SCI control group. (E) Quantification of the relative intensity of VEGF. VEGF expression in the BMMSCs+PEMF group was higher than those in the SCI control, BMMSCs and PEMF group. (F) Quantification of the relative intensity of NGF. There was statistical difference between SCI control and BMMSCs+PEMF group (scale bar 200 μm. Error bars show mean ± SD; *p < 0.05, **p < 0.01, ***p < 0.001, ****p < 0.0001.)

Immunofluorescence was used to investigate the changes in cytokine expression. Compared with the sham group, SCI notably reduced the brain‐derived neurotrophic factor (BDNF) expression. After the different interventions, the expression of BDNF was increased to different degrees but exhibited the highest level in the BMMSCs+PEMF group (Figure 3A and D). Compared with the SCI control group, PEMF group, and BMMSCs+PEMF group prominently increased BDNF expression levels (*p *< 0.05, *p *< 0.01; Figure 3A and D). The expression level of BDNF in the BMMSCs+PEMF group was higher than that in the BMMSCs group (*p *< 0.05). The expression levels of vascular endothelial growth factor (VEGF) in the BMMSCs+PEMF group was significantly increased compared with the SCI control group, BMMSCs group, and PEMF group (*p *< 0.01, *p *< 0.01, *p *< 0.05; Figure 3B and E). Compared with the SCI control group, BMMSCs or PEMF alone did not significantly elevated nerve growth factor (NGF) expression level. Only the combination of these two resulted in a significant increase in NGF level (Figure 3C and F). These results demonstrate that the BMMSCs+PEMF group exhibited the optimal paracrine effects, which were beneficial for SCI recovery.

### 2.3 BMMSCs and PEMF improve the neural functions and axon connections

**FIGURE 4:**
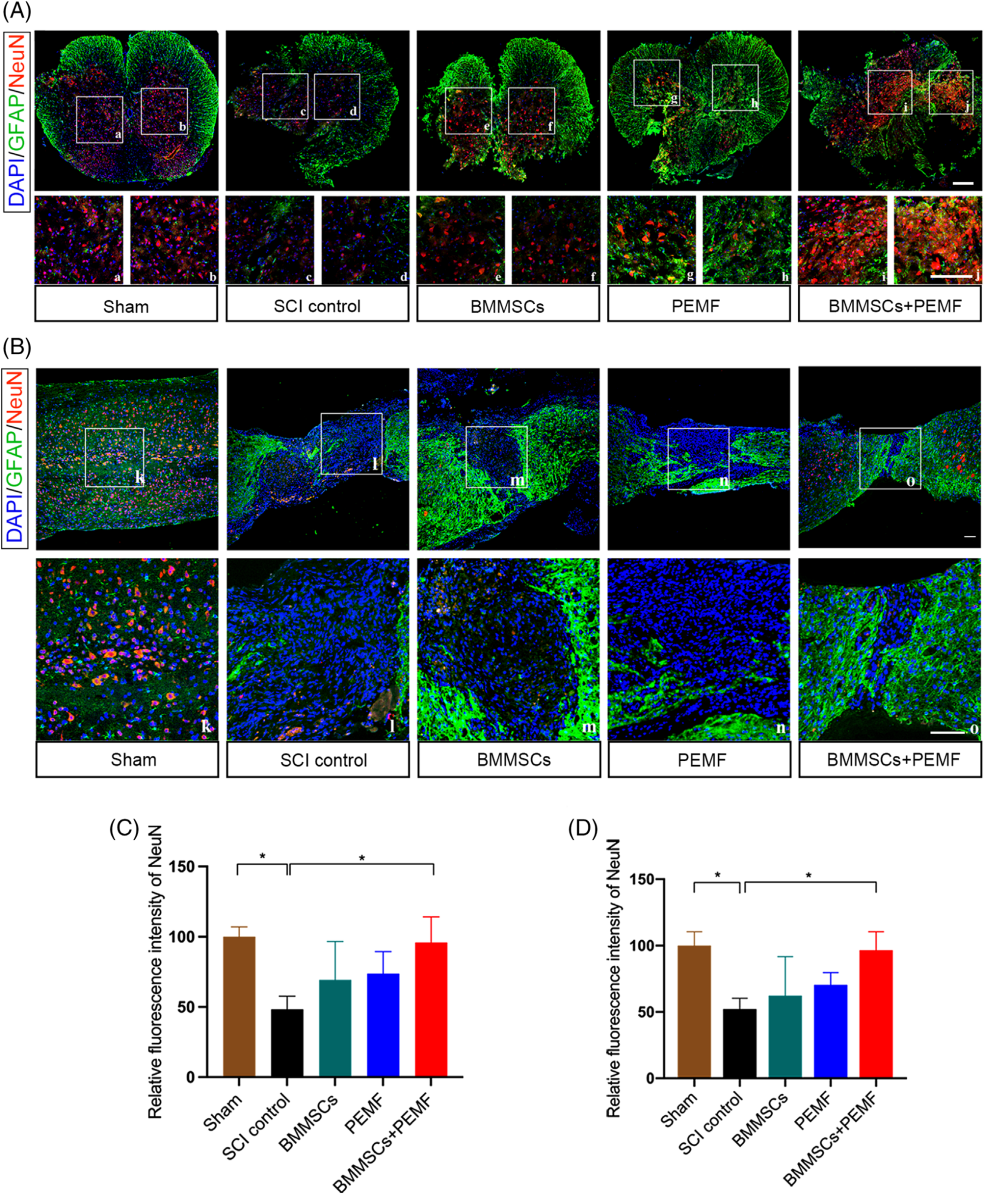
BMMSCs and PEMF promote neuron preservation in vivo. (A) Immunofluorescence staining of astroglial maker GFAP (green), NeuN (red), and DAPI (blue) in spinal cord lesions at week 8 (transverse section). High magnifications of boxed area are displayed below (A–J) (scale bar 100 μm). (B) Immunofluorescence staining of astroglial maker GFAP (green), NeuN (red), and DAPI (blue) in spinal cord lesions at week 8 (longitudinal section). High magnifications of boxed area are displayed below (K–O) (scale bar 200 μm). (C) Quantification of the relative expression levels of NeuN (transverse section). BMMSCs+PEMF significantly enhanced the expression of NeuN after SCI. (D) Quantification of the relative expression levels of NeuN (longitudinal section). BMMSCs+PEMF significantly enhanced the expression of NeuN after SCI. (Error bars show mean ± SD; *p < 0.05.)

**FIGURE 5:**
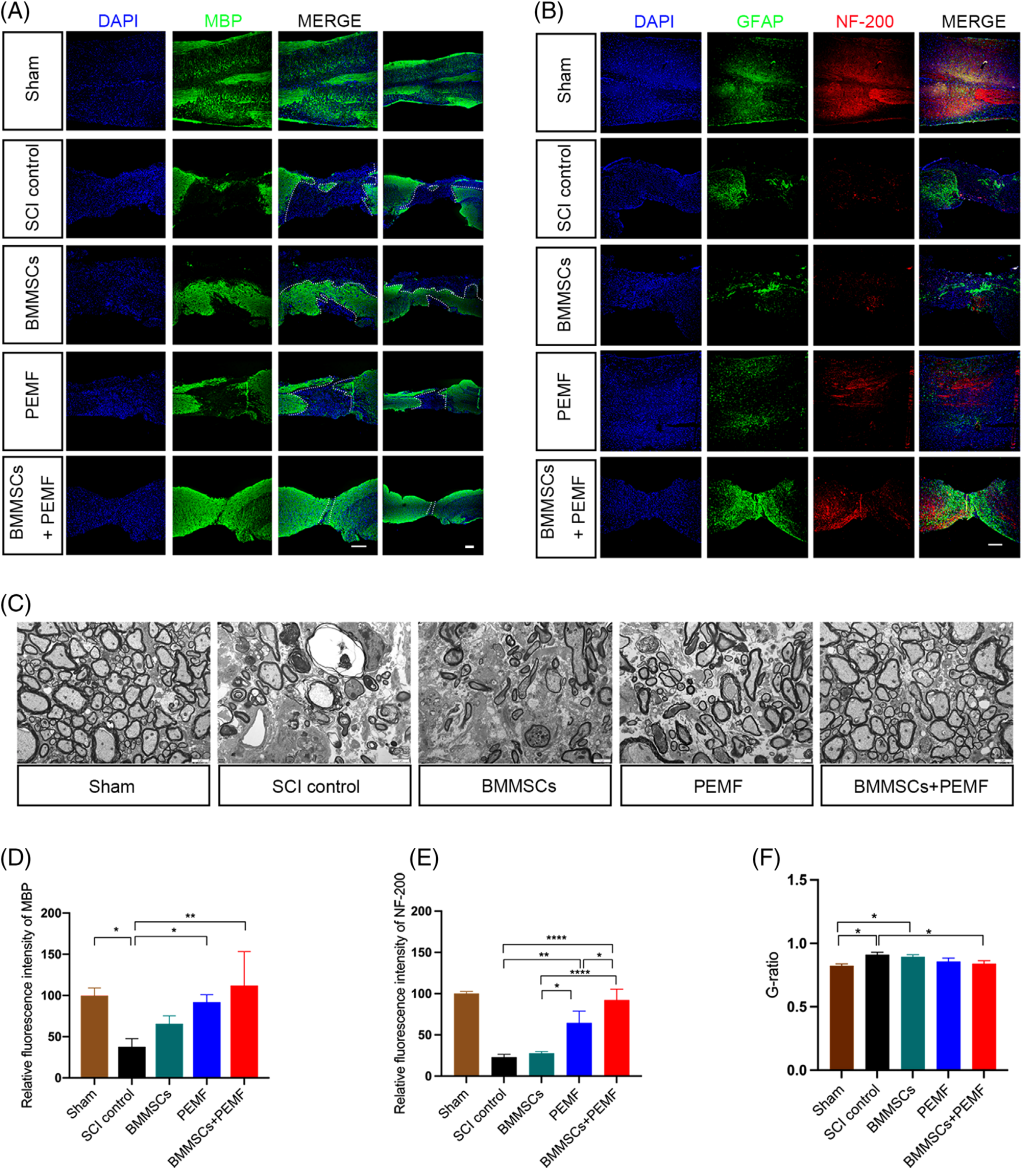
BMMSCs and PEMF alleviate axon destruction and promote neurofilament preservation in vivo. (A) Representative immunofluorescence images of MBP (green) and DAPI (blue) in the sham, SCI control, BMMSCs, PEMF, and BMMSCs+PEMF groups (scale bar 200 μm, left; Scale bar 200 μm, right). (B) Representative immunofluorescence images of NF‐200 (red), astroglial maker GFAP (green) and DAPI (blue) in spinal cord lesions at week 8 (scale bar 200 μm). (C) Representative TEM images of the Sham, SCI control, BMMSCs, PEMF and BMMSCs+PEMF groups (scale bar = 2 μm). (D) Quantification of the relative expression levels of MBP. BMMSCs+PEMF and PEMF increased the expression of MBP. (E) The expression level of NF‐200 in the BMMSCs+PEMF group was significantly higher than those in the SCI control, BMMSCs, and PEMF groups. PEMF also increased NF‐200 expression compared with SCI control and BMMSCs group. (F) Quantitative analysis of G‐ratio values. There was statistical difference between SCI control and BMMSCs+PEMF group. (Error bars show mean ± SD; *p < 0.05, **p < 0.01, ****p < 0.0001.)

We also evaluated the neuronal markers NeuN using immunofluorescence. SCI remarkably reduced the expression levels of NeuN. From the transverse sections, BMMSCs+PEMF significantly increased the expression levels of NeuN compared to the SCI control group (*p *< 0.05; Figure 4A and C). From the longitudinal sections, the expression level of NeuN in the BMMSCs+PEMF group was highest among all injury groups, showing statistical increase compared to SCI control group (*p *< 0.05; Figure 4B and D). Axon integrity was evaluated by MBP immunofluorescence, and the MBP‐positive immunofluorescence signals in the BMMSCs+PEMF group was significantly higher than those in the SCI control group, indicating the best axon integrity condition (*p *< 0.01; Figure 5A and D). PEMF also demonstrated a higher expression level of MBP compared with the SCI control group (*p *< 0.05; Figure 5A and D). The expression levels of neurofilaments maker NF‐200 in the BMMSCs+PEMF group were significantly higher than those in the SCI control, BMMSCs and PEMF group (*p *< 0.0001, *p *< 0.0001, *p *< 0.05), and PEMF also contributed to a remarkable increase of the NF‐200 level compared with the SCI control group and BMMSCs group (*p *< 0.01, *p *< 0.05; Figure 5B and E). The TEM analysis showed less‐dense collagen bundles, fewer degeneration vacuoles and fewer degenerating axons with myelin sheath collapse in the BMMSCs+PEMF group than those in the other injury groups (Figure 5C). G‐ratio was statistically increased after SCI. PEMF slightly reduced G‐ratio while BMMSCs+PEMF obviously reduced it compared with SCI control group (*p *< 0.05, Figure 5F). The TEM outcome further verified the finding of increased myelination and the best axon condition in the BMMSCs+PEMF group.

### 2.4 Combination of BMMSCs and PEMF promotes recovery after SCI through Wnt/β‐catenin signaling pathway

**FIGURE 6:**
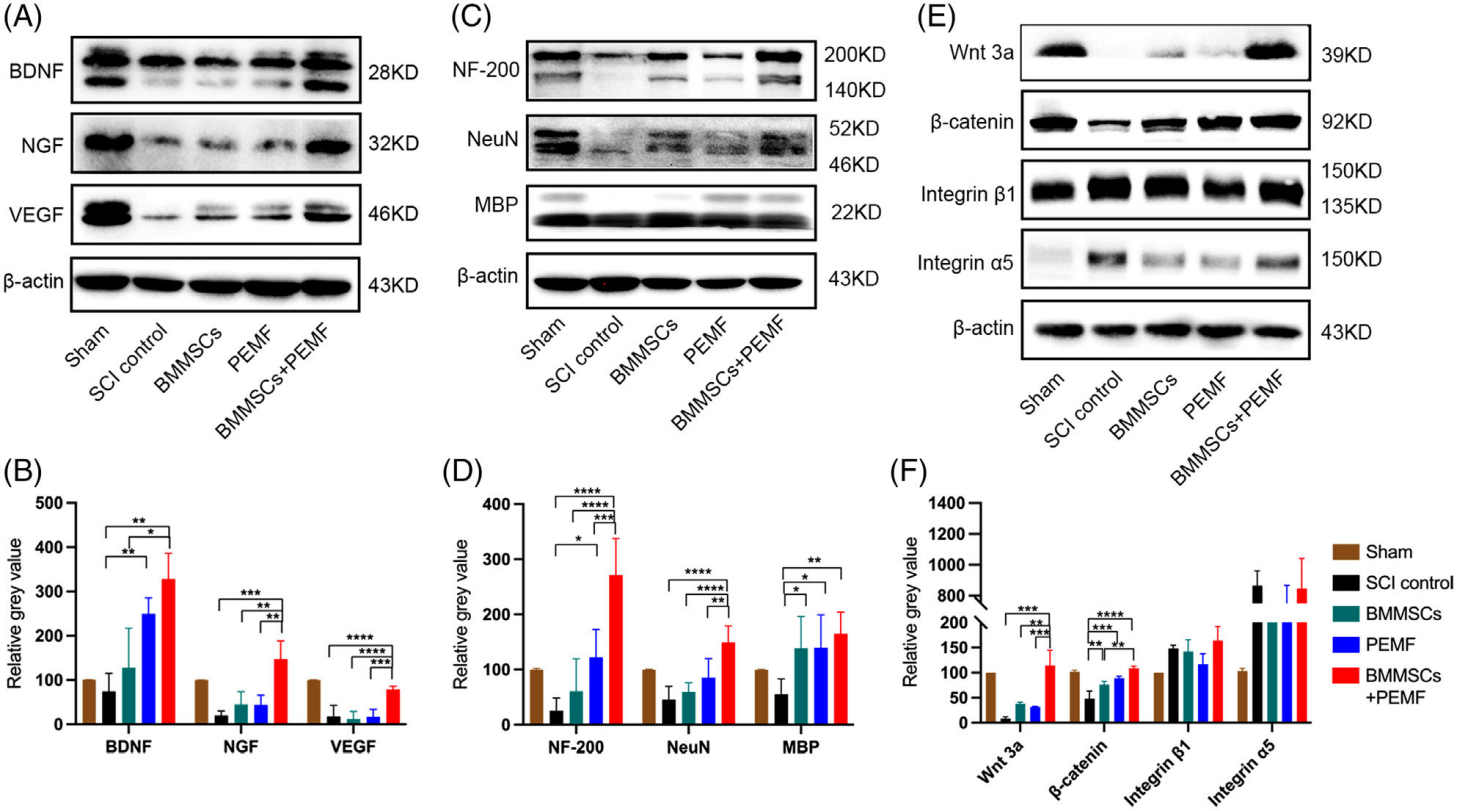
BMMSCs and PEMF increase the expression levels of nutritional cytokines and neuronal markers. (A) The protein levels of BDNF, NGF, and VEGF were investigated by western blotting. GAPDH or β‐actin was used as the loading control. (B) Quantification of the relative expression levels of BDNF, NGF, and VEGF. (C) The protein levels of NF‐200, NeuN, and MBP were analyzed by western blotting. (D) Quantification of the relative expression levels of NF‐200, NeuN, MBP. (E) The protein levels of β‐catenin, integrin β1, and integrin α5 were evaluated by western blotting. (F) Quantification of the relative expression levels of Wnt3a, β‐catenin, integrin β1, and integrin α5. (Error bars show mean ± SD; *p < 0.05, **p < 0.01, ***p < 0.001, ****p < 0.0001.)

Consistent with the immunofluorescence results, BMMSCs+PEMF remarkably increased the protein expression levels of BDNF compared to the SCI control and BMMSCs group (*p *< 0.01, *p *< 0.05; Figure 6A and B). The expression level of NGF in the BMMSCs+PEMF group was statistically increased compared to the SCI control, BMMSCs and PEMF group (*p *< 0.001, *p *< 0.01, *p *< 0.01; Figure 6B). Similarly, the expression level of VEGF was increased in the BMMSCs+PEMF groups compared to the SCI control, BMMSCs, and PEMF groups (*p *< 0.0001, *p *< 0.0001, *p *< 0.001; Figure 6B). The protein expression level of NF‐200 in the BMMSCs and PEMF groups was highest among all injury groups (all *p *< 0.0001), and PEMF also significantly increased the NF‐200 expression compared to SCI control group (*p *< 0.05; Figure 6C and D). In addition, BMMSCs+PEMF obviously increased the protein expression level of NeuN compared with SCI control, BMMSCs, and PEMF groups, respectively (*p *< 0.0001, *p *< 0.0001, *p *< 0.01; Figure 6D). The expression levels of MBP in the BMMSCs, PEMF, and BMMSCs+PEMF groups were increased compared to the SCI control group (*p *< 0.05, *p *< 0.05, *p *< 0.01; Figure 6D). These results indicated that the BMMSCs+PEMF intervention had the best therapeutic efficacy on nerve preservation and axonal function. Previous experimental evidence suggested that the Wnt/β‐catenin signaling pathway is advantageous in the recovery of CNS injury.^32^ To explore whether the Wnt/β‐catenin signaling pathway is involved in the treatment period, the expression of key proteins was determined by western blotting (Figure 6E). The results showed that BMMSCs+PEMF remarkably increased the expression of Wnt‐3a compared with the other interventions administered to the injured groups, respectively (*p *< 0.001, *p *< 0.01, *p *< 0.001; Figure 6F). The expression levels of β‐catenin in the BMMSCs, PEMF and BMMSCs+PEMF groups were significantly increased compared to the SCI control group (*p *< 0.001, *p *< 0.0001, *p *< 0.00001), and BMMSCs+PEMF group also demonstrated significant difference compared with BMMSCs group (*p *< 0.01; Figure 6F). PEMF, BMMSCs and BMMSCs+PEMF did not displayed effect on the expression levels of integrin β1 and integrin α5 (Figure 6F).

### 2.5 PEMF promotes the expression of BDNF and VEGF in BMMSCs in vitro

**FIGURE 7:**
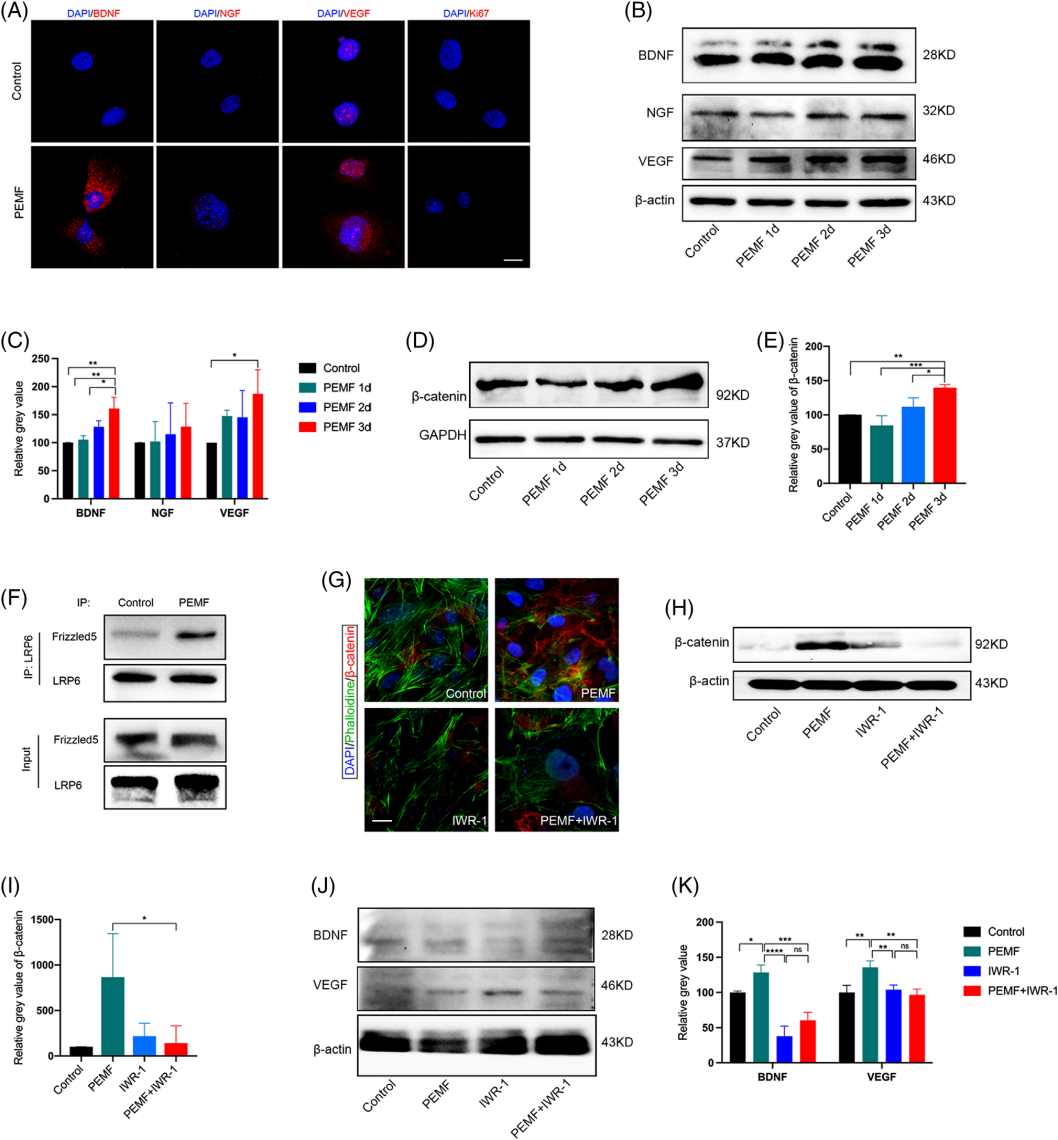
PEMF promotes the expression levels of BDNF and VEGF in BMMSCs in vitro through Wnt/β‐catenin signaling pathway. (A) Representative immunofluorescence images of BMMSCs that were stained for BDNF, NGF, VEGF, and Ki67 (scale bar = 20 μm). (B) The protein levels of BDNF, NGF, and VEGF were analyzed by western blotting. (C) Quantification of the relative expression levels of BDNF, NGF, and VEGF. PEMF 3d group displayed highest expression levels of BDNF among all groups. VEGF was significantly increased in the PEMF 3d group compared with other groups. (D) The protein levels of β‐catenin were analyzed by western blotting. (E) Quantification of the relative expression levels of β‐catenin showed PEMF 3d significantly increased the expression of β‐catenin compared with control group. (F) Co‐IP analysis showed the enhanced interaction between LRP6 and Frizzled5 after PEMF intervention in 293T cells. (G) Representative immunofluorescence images of β‐catenin (red), phalloidine (green), and DAPI (blue) in BMMSCs for all groups. Wnt signaling pathway inhibitor (IWR‐1) significantly inhibited the expression of β‐catenin (scale bar = 20 μm). (H) Wnt signaling pathway inhibitor (IWR‐1) successfully inhibited the expression of β‐catenin. (I) Quantification of the expression levels of β‐catenin. (J) The protein levels of BDNF and VEGF were analyzed by western blotting. (K) The effect of PEMF on the expression of BDNF and VEGF in BMMSCs was partially abolished by a Wnt signaling pathway inhibitor (IWR‐1). (Error bars show mean ± SD; *p < 0.05, **p < 0.01, ***p < 0.001, ****p < 0.0001.)

To explore the effects of PEMF on BMMSCs in vitro, we examined the expression of BDNF, NGF, and VEGF in BMMSCs via immunofluorescence and found that PEMF notably increased the BDNF and VEGF expression levels (Figure 7A). However, PEMF with this parameter did not exhibit a positive effect on the NGF and proliferation (Ki67) of BMMSCs (Figure 7A). The western blot results also revealed a similar tendency. PEMF 3d group obviously increased the expression level BDNF compared with the control, PEMF 1d and PEMF 2d groups (*p *< 0.01, *p *< 0.01, *p *< 0.05; Figure 7B and C). PEMF did not change the expression levels of NGF among all the groups (*p *> 0.05, Figure 7C). PEMF 3d group statistically increased the expression level VEGF compared with the control group (*p *< 0.05; Figure 7C)

### 2.6 PEMF enhances the expression of cytokines of BMMSCs through the Wnt/β‐catenin signaling pathway in vitro

To determine the underlying mechanism of the effects of PEMF on BMMSCs, we evaluated the protein expression of β‐catenin. PEMF 3d group notably increased β‐catenin protein expression levels compared to the control, PEMF 1d and PEMF 2d groups (*p *< 0.01, *p *< 0.001, *p *< 0.05; Figure 7D and E). We also found that LRP6 bound more to Frizzled5 in 293T cells after PEMF intervention (Figure 7F). To clarify the role of Wnt/β‐catenin signaling pathway, cells were pretreated with 1 μM IWR‐1‐endo (Selleck, Houston, TX, #1127442‐82‐3), an inhibitor of this signaling pathway that inhibits β‐catenin accumulation, and then subjected to PEMF intervention. The immunofluorescence results and western blot analysis results both suggested that IWR‐1 successfully suppressed the Wnt/β‐catenin signaling pathway (Figure 7G–I). The inhibition of the Wnt/β‐catenin signaling pathway remarkably abolished the promoting effects of PEMF and decreased the expression levels of BDNF and VEGF (Figure 7J and K), showing that Wnt/β‐catenin signaling pathway was indeed involved in the role of PEMF.

## 3 DISCUSSION

Since BMMSCs alone demonstrate limited therapeutic potential for SCI treatment, there are mounting studies attempt to improve the therapeutic efficiency by means of combinational therapy. Previous studies combined BMMSCs with nutritional cytokines, genetic engineering, other cell types, physical therapy, or biomaterials to promote the efficacy of BMMSCs in the spinal cord injury models, which indeed showed better therapeutic effects compared to single therapy. In the beginning, it is believed that BMMSCs could differentiate into neural cells to repair the injury spinal cord, but the recent consensus suggests that the beneficial effects of BMMSCs on SCI recovery are related to paracrine signaling, cell fusion, and trans‐differentiation instead of differentiation. BDNF is one of the desirable neurotrophic factors owing to its ability to promote synaptic plasticity, survival of exiting neurons, and axonal regeneration.^33^ The therapeutic effects of transplanted BMMSCs in SCI rats are enhanced with the increase of BDNF secretion while the effects are attenuated when the secretion of BDNF is inhibited.^34^ In the present study, we found that PEMF and BMMSCs + PEMF both increased the expression of cytokines BDNF, and the BMMSCs + PEMF was more effective than PEMF alone. NGF is one of the earliest studied neurotrophic factors, which has dual biological functions of neurotrophic and neurite growth promotion. BMMSCs+PEMF increased the expression levels of NGF and VEGF. Our results are consistent with the observations from previous study; that is, the injury site with higher nutritional cytokines is associated with better functional recovery. BMMSCs and PEMF improve the motor functional recovery may be due to increased nutrient cytokines around the injury sites.

Primary injury to spinal cord triggers a series of secondary damage, leading to massive loss of neurons and severe rupture of nerve fibers. Neurons failed to conduct electrical and chemical signals following injury. Protecting neurons and nerve fibers as much as possible is relevant to a reduction of injury volume and recover of spinal cord function. We observed that the expression of neuron‐specific markers NeuN was enhanced in the BMMSCs+PEMF group, which displayed the neuroprotection effects of BMMSCs and PEMF. Although there were some studies demonstrated that BMMSCs could differentiated into neurons,^35^ we speculated that the combinational therapy of BMMSCs and PEMF facilitated neuroprotection instead of neuronal differentiation. Additionally, neurofilaments were better preserved both in the PEMF group and BMMSCs+PEMF group. In addition to the protective effect of combination therapy on neurofilaments, PEMF also exhibited the certain effect on it; this may be because PEMF can promote regeneration after neural impairment.^15^

Axonal damage and demyelination seriously impede SCI recovery; therefore, inhibition of demyelination and promotion of axonal regeneration are beneficial to SCI recovery. BMMSCs+PEMF and PEMF both increased the expression of MBP, and the combinational therapy displayed the better protective effect than PEMF alone. It is reported that PEMF had a significant effect on neurite outgrowth,^36^ which may be the reason of the effect of PEMF on MBP preservation. According to the TEM results, the thickness of myelin sheath is highest in the BMMSCs+PEMF group among all the groups, suggesting that the improved functional recovery of this group may be attributed to the preserved myelin sheath around the injury site. In addition, oligodendrogenesis and myelination have been hypothesized to be beneficial to motor function recovery,^37^ and the functional recovery in the PEMF group and BMMSCs+PEMF group appears to be attributed to the attenuation of secondary injury damage to axons. Given that regular horizontal ladder task evaluates the skill locomotor function and it is more suitable for mice with frequent or consistent plantar stepping, meaning a BMS score of 5 is required^38^; therefore, it is reasonable that only BMMSCs+PEMF reduced the error rate of hindlimbs in the regular horizontal ladder analysis.

Canonical Wnt signaling pathway enhanced axonal regeneration and neurite growth, indicating that exogenous regulation of Wnt may work as a desirable target for SCI recovery.^32^ Researchers also investigated the neurogenesis effect of the noncanonical Wnt signaling pathway and found that these pathway proteins are advantageous for recovery after CNS injury.^32^ In accordance with previous results, we found that BMMSCs+PEMF activates β‐catenin, the key protein of Wnt/β‐catenin signaling pathway. The activation of Wnt/β‐catenin signaling pathway plays a pivotal role in the neuronal and axonal protection, thereby augmenting motor functional recovery. Besides, the canonical Wnt signaling pathway and integrin β1 also induce astrocyte polarization and play pivotal roles in the extension of long protrusions towards the injury site.^39^ However, there was no significant change in the expressions of integrin α5 and integrin β1 in the present study. Notch signaling pathway suppresses neuronal differentiation and astrocyte lineage differentiation in neural stem cells.^40^, ^41^ Suppression of the Notch signaling pathway exhibits a positive effect on neuronal differentiation both in animals and cells experiments.^42^, ^43^ Components of the Wnt/β‐catenin signaling and Notch pathways engage in cross talk. Wnt/β‐catenin upregulates the expression of the proneural transcription factor neurogenin1 (Ngn1) and neuronal differentiation factor 1 (NeuroD1), which are inhibited by Hes1 and Hes5 in the Notch signaling pathway. We found PEMF, BMMSCs, and PEMF+BMMSCs groups slightly downregulated the expression level of Notch but these differences were not statistical (data not shown), suggesting that the Notch signaling pathway was not involved in the synergetic effects of PEMF and BMMSCs.

To investigate the potential interaction between the PEMF and BMMSCs in vitro, we used PEMF intervention on BMMSCs with different duration in vitro. We observed that the expression levels of BDNF and VEGF in BMMSCs increased after PEMF intervention, which is one of the reasons for the best effect in the combined treatment group. We also observed that PEMF upregulated the expression of β‐catenin, suggesting the activation of Wnt/β‐catenin signaling pathway after PEMF intervention. In addition, the current view is that the canonical Wnt/β‐catenin signal transduction requires proximity of LRP5/6 to Frizzled coupled by Wnt ligands.^44^ It is difficult for LRP5/6 and Frizzled to be simultaneously captured by Wnt ligands when they are far apart. We found direct interaction between LRP6 and Frizzled5, which further confirmed the function of the Wnt/β‐catenin pathway. We further explored the role of the Wnt/β‐catenin signaling pathway in cytokine expression using an inhibitor of the Wnt/β‐catenin signaling pathway (IWR‐1). IWR‐1 partially counteracted the promoting effects of PEMF on cytokine expression, indicating that the canonical Wnt/β‐catenin signaling pathway was involved in the effects of PEMF on BMMSCs.

Similarly, researchers have demonstrated that pretreating BMMSCs with low intensity pulsed ultrasound (LIPUS) stimulation enhanced the therapeutic efficiency of BMMSCs than untreated BMMSCs in SCI.^45^ LIPUS promoted the proliferation and decreased the apoptosis of BMMSCs. Additionally, LIPUS enhanced the expression of BDNF, NGF, and NSE (neuronal differentiation markers). In that study, LIPUS altered the characteristics and improved the therapeutic effect of BMMSCs but did not play a direct role in SCI recovery. In our study, PEMF not only strengthened the efficiency of transplanted BMMSCs but also exhibited direct useful effects on SCI. According to the BMS score, the functional gains of the BMMSCs+PEMF group were significantly greater than those of the other three groups, which was attributed to the combined effect of BMMSCs and PEMF. In brief, combination of BMMSCs and PEMF exhibited the most satisfactory therapeutic effects than the single therapy.

There are some limitations to this study. First, survival after stem cell transplantation cannot be tracked because the BMMSCs are not labeled. Second, considering the therapeutic effects of combination therapy is multifaceted, the mechanism of different benefit of combination therapy needs to be further explored.

## 4 CONCLUSION

In summary, combination of PEMF and BMMSCs enhanced functional recovery in SCI mice by improving the expression of BDNF, NGF, and VEGF, promoting neuron preservation and axon growth, which may be achieved by modulating Wnt/β‐catenin signaling pathway. Besides, PEMF also enhanced the expression levels of BDNF and VEGF of BMMSCs in vitro via Wnt/β‐catenin signaling pathway. The synergistic effect of PEMF and BMMSCs enhanced locomotor functional recovery, offering tremendous hope for effective SCI treatment. Continued study on optimization of the treatment effects and exploration into the underlying mechanisms are necessary.

## 5 MATERIALS AND METHODS

### 5.1 Isolation and identification of BMMSCs

C57BL/6 mice (female, 3–5 weeks, 14–16 g) were purchased from Chengdu Dossy Experimental Animals Co., Ltd. All animal studies were conducted in accordance with the ethics guidelines of the Animal Ethics Committee of the West China Hospital of Sichuan University. All animals were housed on a 12 h light and 12 h dark cycle and free to eat and drink. C57BL/6 mice were sacrificed by cervical vertebral luxation after anesthetization and then transferred to 75% ethyl alcohol for a few minutes. The femur and tibia were removed using surgical scissors and surgical forceps. Residual muscle and fascia were removed as much as possible before the bone marrow cavity was exposed. Then, the ends of the femur and tibia were cut with surgical scissors. Then, the bone marrow cavity was rinsed with MEM Alpha basic (Gibco, Grand Island, NY, #C12571500BT) containing 10% FBS (Gibco, Grand Island, NY, #10099‐141) and 1% penicillin‐streptomycin (Gibco, Grand Island, NY, #15140‐122) until it turned pale. The cell suspension was cultured in an incubator at 37°C and 5% CO_2_. After 48–72 h, cells were gently washed with PBS, and the culture medium was refreshed for the first time. Thereafter, the medium was changed every three days until the density of the cells reached 80%–90%.^31^ Then, the cells were digested with trypsin (Gibco, Grand Island, NY, #25200‐056) for 2 min and passaged at 1:3. Passages 3–6 were used for our experiments.

Cells were digested with trypsin, washed twice with staining buffer (1:200, BD Bioscience, Franklin Lake, NJ, #554656) and coincubated with fluorescently labeled primary antibodies against CD29 (1:200, BD Bioscience, Franklin Lake, NJ, #562801), CD34 (1:200, BD Bioscience, Franklin Lake, NJ, #553733), CD44(1:200, BD Bioscience, Franklin Lake, NJ, #559250), and CD45 (1:200, BD Bioscience, Franklin Lake, NJ, #550994) for half hour on ice. Next, the cells were centrifuged twice at 500 × *g* for 5 min and resuspended in staining buffer. Anti‐CD29, anti‐CD34, anti‐CD44, and anti‐CD45 were identified by Cytoflex (Beckman, IN, USA). Osteogenic differentiation of stem cells was induced by osteogenic induction medium (complete medium with 50 μg/ml ascorbic acid, and 10 mmol/L β–glycerophosphate). Medium was replaced every third day and Alizarin red staining was used to observe the osteogenic differentiation of cells after 21 days.

### 5.2 SCI model and BMMSCs transplantation

C57BL/6 mice (female, 6–8 weeks, 18–20 g) were used for animal experiments. Pentobarbital sodium (40 mg/kg) was used for anesthesia for 75 mice and 2% isoflurane was used to maintain the anesthetic effect. The back skin was shaved with a shaver and then sterilized by iodophor. Then, the back skin was cut with a scalpel, and the muscle and fascia overlying the lamina were cleaned. Mice were subjected to laminectomy to expose the dorsal portion of the spinal cord corresponding to the T10 levels. Then the contusion injury was performed at T10 level with an Infinite Horizon (IH) impactor (Precision Systems and Instrumentation, Lexington, KY, USA) at 70 kilodynes. Hindlimb spasms and flicking of the tail were considered as signs of successful impact. Sixty mice were randomly assigned to four groups: SCI control group, BMMSCs group, PEMF group, and BMMSCs+PEMF group (*n* = 15 per group). Mice in the sham groups (*n* = 15) received laminectomy but no injury to the spinal cord. In the BMMSCs group and BMMSCs+PEMF group, the injury sites of the mice were injected with 1 × 10^6^ BMMSCs^46^ in 5 μl of medium using a Hamilton microsyringe and infusion pump (RWD Life Science CO., LTD, Shenzhen, China). The injection rate was 1 μl/min, and the microsyringe was left for a few minutes after injection. Muscle and skin layers were sutured with 4‐0 absorbable suture. Penicillin was injected intraperitoneally for 3 days (50,000 U/kg/d), and the bladder was manually evacuated every day until bladder function was restored.

### 5.3 PEMF intervention in vivo and in vitro

The magnetic field device was manufactured by Sichuan University. The device consists of three parts: a pulse generator, a stepper motor driver, and a Helmholtz coil.^47^ A 1000‐turn copper wire constitutes a cylindrical solenoid. The coil provides stable magnetic field in the center area. Mice of the PEMF group (*n* = 15) and BMMSCs+PEMF group (*n* = 15) were exposed to PEMF for 8 weeks from the first day after the injury. The frequency, intensity and duration were 50 Hz, 1 mT, 1 h/day. The intervention parameters were followed previous study with slight modifications.^48^ Mice and cages were placed in the PEMF instrument for intervention. Mice in the sham group (*n* = 15), SCI control group (*n* = 15), and BMMSCs group (*n* = 15) received sham PEMF for same time without any output. After PEMF intervention, the mice were transferred back to their room.

Another magnetic field device was installed in a cell incubator for PEMF intervention in vitro. BMMSCs were exposed to PEMF, and the frequency and intensity (50 Hz, 1 mT) were the same as PEMF intervention in vivo. The intervention time was 3 h per day.

### 5.4 Behavioral assessments

The Basso Mouse Scale (BMS) is commonly used to measure motor capacity of contused SCI mice due to its sensitivity, effectiveness, and reliability.^49^ It is an open‐field locomotor evaluation test ranging from 0 points, representing no ankle movement, and 9 points, representing approximate normal motor function (Table S1). The BMS primarily evaluates hindlimb function, trunk stability, and tail position. Final score was averaged between left and right limbs. The BMS was assessed by two investigators independently during a 3‐min period.

#### 5.4.1 Horizontal regular ladder analysis

The horizontal ladder consists of 70 rungs spaced 1.3 cm apart at 30 cm height.^38^ Mice were recorded with a high‐definition mobile phone crossing of 30 rungs. Success steps represent plantar or toe placements on the rung, and error steps represent slipping off the rung, missing the rung and dragging on the rung. Mice were re‐recorded if they stop for a few seconds or return in the direction they started. Number of success and errors were recorded. Error rate was expressed as percentage of the number of error steps in the total number of the steps. This analysis was performed and analyzed every week.

#### 5.4.2 Footprint analysis

The plantar surface of four limbs of the mice was colored by nontoxic ink (red for hindlimbs and blue for forelimbs). Then the mice walk in a narrow corridor padded with a white sheet of paper at the bottom for three times. The plantar stepping and hindlimb base of support of the mice were observed.

### 5.5 Magnetic resonance imaging

A small animal 7T magnetic resonance imaging system (BioSpec 70/30, Bruker, Karlsruhe, Germany) was used to evaluate the extent of the injury by volume. The mice were anesthetized with an isoflurane/oxygen mixture (3% isoflurane for induction and 1.5% isoflurane for maintenance). Then, the mice were placed on a fixation device in a prone position and scanned. The MRI parameters were as follows: T2‐weighted; 256 × 256 matrix; slice thickness, 1 mm; intersection gap, 1 mm; echo time/repetition time, 27/3000 ms. The injury area was measured using ImageJ software (v1.52, NIH, USA).

### 5.6 Masson staining

For paraffin sections, mice were subjected to cardiac perfusion with precooling phosphate buffer saline (PBS) and 4% paraformaldehyde; injured spinal cords were collected and fixed in 10% formaldehyde, embedded in paraffin, and sliced longitudinally into 2.5 μm sections. For frozen sections, the spinal cord was collected after perfusion, fixed in the 4% paraformaldehyde overnight at 4°C and then dehydrated in different concentrations of sucrose solution. Tissue was embedded with Tissue‐Tek® O.C.T. Compound (SAKURA, USA, 4583) and sliced transversely into 10 μm sections, stored at –20°C for further use. Masson staining was performed according to the manufacturer's instructions (Solarbio, Beijing, China). Sections were dewaxed and rehydrated regularly, stained with Weigert iron brazilwood staining fluid for 5–10 min, differentiated with acidic ethanol liquid for 5–15 s, and washed with distilled water. Then, the sections were treated with Masson blue fluid for 3–5 min, washed with distilled water for 1 min and stained with ponceau for 5–10 min. Next, the sections were washed with weak acid working liquid for 1 min, washed with phosphomolybdic acid solution for 1–2 min and washed with configured weak acid working liquid for 1 min. Then the sections were stained with aniline blue staining solution for 1–2 min and washed with the prepared weak acid working solution for 1 min again.^50^ The sections were rapidly dehydrated with 95% ethanol and then dehydrated with anhydrous ethanol 3 times for 5–10 s each time, cleared with xylene 3 times and sealed with neutral resin. Images were collected using Ni‐E (Nikon, Tokyo, Japan) and analyzed with NIS analysis software.

### 5.7 Nissl staining

Frozen sections were thawed at room temperature for 1 h and rehydrated in PBS, dyed in Nissl staining solution at room temperature for 10–20 min, dehydrated with gradient alcohol, soaked in xylene, and sealed with neutral resin. Nissl bodies were detected using an Ni‐E system (Nikon, Tokyo, Japan) and analyzed with NIS analysis software.

### 5.8 Immunohistochemistry

The paraffin sections were dewaxed and rehydrated, followed by antigen retrieval using citrate antigen retrieval solution (pH = 6.0) with 15 min heating. When the sections were naturally cooled to room temperature, they were treated with 3% hydrogen peroxide to eliminate the influence of endogenous peroxidase. Then, the sections were washed with PBS three times, blocked with 10% goat serum (Zsbio, Beijing, China, ZL9021) for 15 min at 37°C, and incubated with primary antibodies overnight at 4°C. Primary antibodies were diluted as follows: GFAP (1:300, CST, #3670), BDNF (1:100, Proteintech #28205‐1), NGF (1:100, Abcam, ab6199), VEGF (1:250, Proteintech, #19003‐1), NeuN (1:100, Proteintech, #26975‐1), MBP (1:100, Proteintech, #10458‐1), NF‐200 (1:100, Proteintech, #18934‐1), Ki67 (1:100, Beyotime, #AF1738), β‐catenin (1:200, Proteintech, #51067‐2). The next day, sections were rinsed with PBS three times and incubated with corresponding secondary fluorescent antibodies rhodamine (TRITC)‐conjugated AffiniPure goat anti‐rabbit IgG (H+L) (Jackson ImmunoResearch, West Grove, PA, #111‐025‐003) and/or CoraLite488‐conjugated AffiniPure goat anti‐mouse IgG (H+L) (Proteintech, Wuhan, China, #SA00013‐1) for 1 h at room temperature. Nuclei were counterstained with 4,6‐diamidino‐2‐phenylindole (DAPI) (Biosharp, Hefei, China, #BL105A). Then the samples were mounted with clear liquid mounting medium (Southern Biotech, Birmingham, AL, #0100‐01). Frozen sections were thawed at room temperature for 1 h and rehydrated in PBS. After permeabilized with 0.5 % Triton X‐100, sections were blocked with 10% goat serum for 15 min at 37°C and the following steps were the same as above. Fluorescence signals were observed using a confocal laser scanning microscope N‐STORM & A1 (Nikon, Tokyo, Japan) and analyzed with NIS analysis software.

### 5.9 Transmission electron microscopy (TEM)

To investigate the ultrastructure of the spinal cord, the mice were subjected to cardiac perfusion using 2.5% glutaraldehyde, and spinal cord tissue was quickly fixed in 3% glutaraldehyde and then fixed with 1% osmium tetroxide. Then, the tissues were dehydrated with acetone step by step. The dehydrated samples were successively passed through the dehydrating agent and epoxy resin osmotic solution. The permeable samples were placed into the appropriate mold, infused with embedding liquid, and heated to form a solid matrix (embedded block). Ultrathin sections approximately 50‐nm thick were prepared by the ultrathin section mechanism, floated on the liquid level of the knife groove, and then transferred to copper mesh. Sections were first stained using uranium acetate for 10–15 min, then stained with lead citrate for 1–2 min at room temperature, and subsequently observed by transmission electron microscopy with a JEM‐1400PLUS. G‐ratio represents the proportion of axon diameter to the entire nerve fiber diameter (axon and myelin sheath).

### 5.10 Western blotting

The injured spinal cords of the mice were collected after cardiac perfusion and stored in liquid nitrogen immediately. The tissue was lysed with radioimmunoprecipitation assay (RIPA) lysis buffer (Beyotime, Shanghai, China), homogenized using a tissue homogenizer (Tissuelyser‐48, Jingxin, Shanghai, China), and centrifuged for 15 min (12,000 × *g*) at 4°C. The protein samples were subjected to sodium dodecyl sulfate‐polyacrylamide gel electrophoresis (SDS‐PAGE) and transferred to polyvinylidene fluoride (PVDF) membranes. Then, the membranes were blocked with 5% nonfat milk for 1 h at room temperature and washed three times using Tris‐buffered saline plus Tween (TBST). Next, the membranes were incubated overnight with homologous primary antibodies at 4°C. Primary antibodies were diluted as follows: BDNF (1:1000, Proteintech #28205‐1), NGF (1:1000, Abcam, ab6199), VEGF (1:1000, Proteintech, #19003‐1), NeuN (1:1000, Proteintech, #26975‐1), MBP (1:1000, Proteintech, #10458‐1), NF‐200 (1:1000, Proteintech, #18934‐1), β‐catenin (1:5000, Proteintech, #51067‐2), Wnt 3a (1:1000, Bioss, bs‐23278R), Integrin β1 (1:1000, CST, #34971T), Integrin α5 (1:1000, CST, #4705T), β‐actin (1:10000, Abways, #AB2001), and GAPDH (1:10000, Abways, #AB2000). The next day, the membranes were washed with TBST and then incubated with horseradish enzyme‐labeled goat anti‐rabbit IgG (Zsbio, Beijing, China, #ZB‐2301) at room temperature for 1 h. Images were obtained using Immobilon western chemiluminescent HRP substrate (Millipore, Darmstadt, Germany) and a chemiluminescence imager (Bio‐Rad, CA, USA). Relative gray values were analyzed using ImageJ software.

### 5.11 Immunofluorescence

BMMSCs were washed with PBS three times and fixed in 4% paraformaldehyde for 30 min at room temperature. Then cells were blocked in 10% goat serum for 30 min at 37°C and incubated primary antibodies overnight at 4°C. Primary antibodies were diluted as previous description. The next day, cells were placed for 30 min at room temperature and washed with PBS three times. Next, cells were incubated with corresponding secondary fluorescent antibodies rhodamine (TRITC)‐conjugated AffiniPure goat anti‐rabbit IgG (H+L) and/or CoraLite488‐conjugated AffiniPure goat anti‐mouse IgG (H+L) for 1 h at room temperature. Nuclei were counterstained with DAPI. Then the samples were mounted with clear liquid mounting medium. Fluorescence signals were observed using a confocal laser scanning microscope N‐STORM & A1 (Nikon, Tokyo, Japan) and analyzed with NIS analysis software.

### 5.12 Coimmunoprecipitation

Cells were lysed with IP lysis buffer (Beyotime, Shanghai, China) containing protease inhibitor cocktail and phosphatase inhibitors for 30 min. The lysates were centrifuged at 12,000 × *g* for 10 min at 4°C. the supernatants mixed with 1× loading buffer were boiled at 100°C for 10 min as input. Protein A/G Magnetic Beads (MCE, Cat# HY‐K0202) were washed with wash buffer for 4 times and 400 μl of diluted antibody (LRP6, 1:50, CST, #3395) were added to the Protein A/G Magnetic Beads. The mixture in the tubes were rotated at 4°C overnight and then washed with wash buffer for 4 times. Next, the supernatants were added to the Protein A/G Magnetic Beads‐Antibody complex and incubated with rotation at 4°C for 2 h. The final supernatants were collected after magnetic separation and used for denaturing SDS‐PAGE.

### 5.13 Statistical analysis

Data are shown as the mean ± SD. GraphPad Prism Software version 8.0.0 (131) (San Diego, CA) was used for statistical analyses. The method of statistical analysis was one‐way analysis of variance (ANOVA) with Tukey's multiple comparison post hoc test. Two‐way ANOVA Tukey's multiple comparison post hoc test was used for the BMS score and horizontal ladder analysis. *p* < 0.05 was considered statistically significant.

## CONFLICT OF INTEREST

The authors declare no conflict of interest.

## AUTHOR CONTRIBUTIONS

L.H. and Q.W. designed the research and prepared the manuscript. L.H., X.S., L.W., G.P., Y.W., Q.Z., Z.L., D.W., and C.F. performed the research. C.H. provided advice for the research. Q.W. revised the manuscript and approved the final version of the manuscript.

## ETHICS APPROVAL

All mouse experiments and care were performed in accordance with a procedure approved by the Animal Ethics Committee of the West China Hospital, Sichuan University (Approval No. 2020292A).

## Supporting information

Supporting Information

## Data Availability

The data sets generated and analyzed during the current study are available from the corresponding author upon reasonable request.
