# Supplementary material for: Enhanced effect of combining bone marrow mesenchymal stem cells (BMMSCs) and pulsed electromagnetic fields (PEMF) to promote recovery after spinal cord injury in mice
Source: MedComm (2020). 2022 Aug 3;3(3):e160. doi: 10.1002/mco2.160 (PMC9350428; doi:10.1002/mco2.160)
Supplement: Supplementary file 1 — Supporting Information [file MCO2-3-e160-s001.docx]

**Enhanced effect of combining Bone marrow mesenchymal**

**stem cells (BMMSCs) and pulsed electromagnetic fields (PEMF)**

**to** **promote recovery after spinal cord injury in mice**

Liyi Huang^1,2^, Xin Sun^1,2^, Lu Wang^1,2^, Gaiqing Pei^1,2^, Yang Wang^1,2^, Qing Zhang^1,2^, Zejun Liang^1,2^, Dong Wang^1,2^, Chenying Fu^3,4^, Chengqi He^1,2^, Quan Wei^1,2*^

^1^ Rehabilitation Medicine Center and Institute of Rehabilitation Medicine, West China Hospital, Sichuan University, Chengdu, PR China.

^2^ Key Laboratory of Rehabilitation Medicine in Sichuan Province, Sichuan University, Chengdu, PR China.

^3^National Clinical Research Center for Geriatrics, West China Hospital, Sichuan University, Chengdu, Sichuan, PR China

^4^Aging and Geriatric Mechanism Laboratory, West China Hospital, Sichuan University, Chengdu, Sichuan, PR China

Article type: original article

*Correspondence to Dr. Quan Wei, E-mail: [weiquan@scu.edu.cn](mailto:weiquan@scu.edu.cn), Tel: 86-02885422847

Rehabilitation Medicine Center and Institute of Rehabilitation Medicine, West China Hospital, Sichuan University, Chengdu, Sichuan, PR China.

Key Laboratory of Rehabilitation Medicine in Sichuan Province, Sichuan University, Chengdu, PR China.

**TABLE S1. SCORES AND OPERATIONAL DEFINITIONS FOR THE BASSO MOUSE SCALE FOR LOCOMOTION (BMS)**

| *Score* |
| --- |
| 0 No ankle movement |
| 1 Slight ankle movement |
| 2 Extensive ankle movement |
| 3 Plantar placing of the paw with or without weight support -OR- Occasional, frequent or consistent dorsal stepping but no plantar stepping |
| 4 Occasional plantar stepping |
| 5 Frequent or consistent plantar stepping, no coordination -OR-Frequent or consistent plantar stepping, *some* coordination, paws *rotated* at initial contact and lift off (R/R) |
| 6 Frequent or consistent plantar stepping, *some* coordination, paws *parallel* at initial contact (P/R, P/P) -OR-Frequent or consistent plantar stepping, *mostly* coordinated, paws *rotated* at initial contact and lift off (R/R) |
| 7 Frequent or consistent plantar stepping, *mostly* coordinated, paws *parallel* at initial contact and *rotated* at lift off (P/R) -OR-Frequent or consistent plantar stepping, *mostly* coordinated, paws *parallel* at initial contact and lift off (P/P), and *severe* trunk instability |
| 8 Frequent or consistent plantar stepping, *mostly* coordinated, paws *parallel* at initial contact and lift off (P/P), and *mild* trunk instability -OR-Frequent or consistent plantar stepping, *mostly* coordinated, paws *parallel* at initial contact and lift off (P/P), and *normal* trunk stability and tail *down or up* & *down* |
| 9 Frequent or consistent plantar stepping, *mostly* coordinated, paws *parallel* at initial contact and lift off (P/P), and *normal* trunk stability and tail *always* up. |


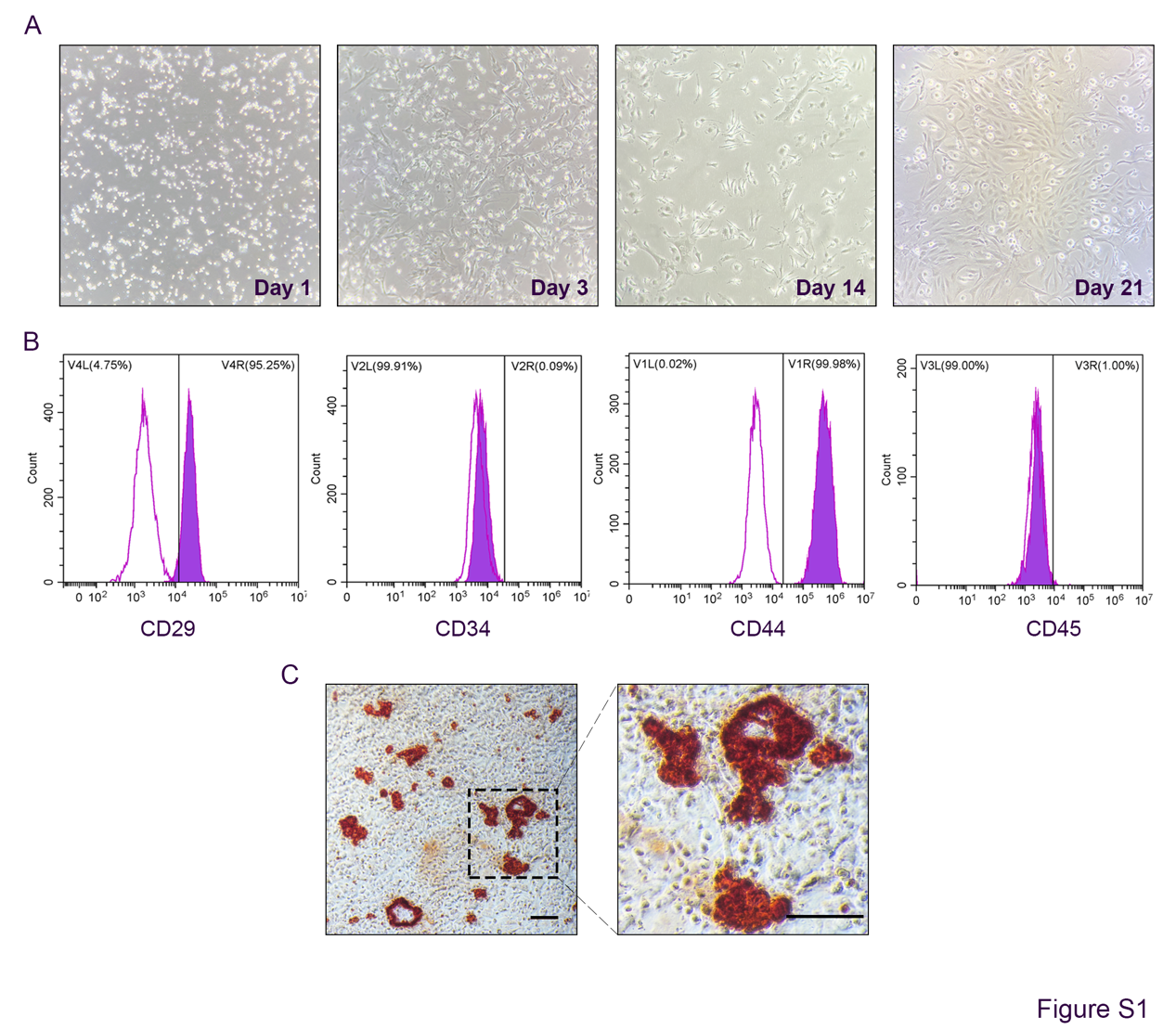


**Figure S1 Cell morphology and characteristics of bone marrow mesenchymal stem cells.**

(A) The cell morphology of BMMSCs 1, 3, 14, and 21 days after primary isolation (magnification ×200).

(B) Cell surface markers (CD29, CD34, CD44 and CD45) were detected by flow cytometry. The percentages of the surface markers CD29, CD34, CD44, and CD45 were 95.25%, 0.09%, 99.98%, and 1.00%, respectively.

(C) Alizarin red staining indicated that BMMSCs have the ability of osteogenic differentiation (scale bar 100 µm).
